# Human minus Three Pieces of Hair

**DOI:** 10.3201/eid1810.AC1810

**Published:** 2012-10

**Authors:** Polyxeni Potter

**Affiliations:** Centers for Disease Control and Prevention, Atlanta, Georgia, USA

**Keywords:** art science connection, emerging infectious diseases, art and medicine, Human minus Three Pieces of Hair, Mori Sosen, Monkey Performing the Sanbasō Dance, macaques, reservoirs, disease transmission, about the cover

**Figure:**
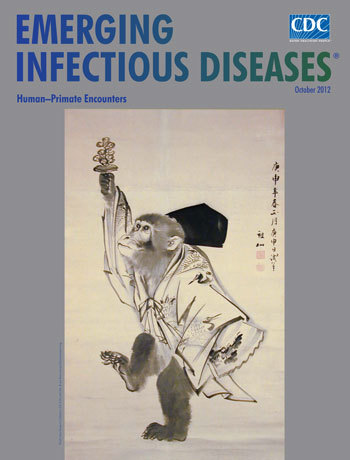
**Mori Sosen (1747–1821) *Monkey Performing the Sanbasō Dance* (Dated 1800, the first day of the Monkey Year) Scroll painting, ink on paper (49.5 cm × 115.6 cm)** Pacific Asia Museum Collection, Gift of Mr. and Mrs. Bruce Ross www.pacificasiamuseum.org

At age 61, Mori Sosen changed the first character of his name to one meaning “monkey.” So close had he become to the subject of his paintings. To learn how to paint the animals convincingly, he lived for a time in the mountains, their natural environment, not relying as others before him on copying their images from Chinese art. His mastery in depicting the Japanese macaque earned him the title “undisputed master” from Dutch orientalist Robert van Gulik (1910–1967). In his book, Gibbon in China, van Gulik translated Confucian scholar Kimura Kenkadō’s account of the animal’s arrival in Japan. “In the winter of the sixth year of the era (1809), a gibbon was shown in Osaka …. Although we have heard the word ‘gibbon’ since olden times and seen pictures of him, we never had seen a live specimen, and therefore a large crowd assembled to see this gibbon. Generally he resembled a large macaque, and figure and fur are very similar.” Mori Sosen created a graphic record of this sensational event.

Not much is known about his life but that he grew up with and around artists, started his training with his father Mori Jokansai, and lived most of his life in Osaka. Mori Sosen’s legacy is his painting of animals, particularly monkeys, their personalities and attitudes as well as their coats and the movement of their muscles underneath. So well did he depict the nature of monkeys that he was accused of being their descendent. He established, with his brother Shūhō, a school of animal painting along the lines of the *Maruyama-Shijō* school in Kyoto. Shūhō’s son studied there under Maruyama Ōkyo, an expert in a style influenced by Western realism and direct observation. The *Shijō* school promoted synthesizing this modern development with the local trend toward the decorative and stylized.

*Monkey Performing the Sanbasō Dance*, on this month’s cover, showcases both Mori Sosen’s favorite subject matter and his artistic style, a blend of realism and expressiveness. The action is set against a vacant background, the viewer drawn toward the main figure. Bold deliberate strokes outline the facial features, right hand and both feet of the animal, and folds in the kimono. Smudged strokes from a dry brush draw the ruffled texture of the fur against the black cap and smooth, mostly unpainted surface of the clothing. The monkey, mouth pursed with concentration, eyes fixed on some point outside the painting, holds a fan in one hand, and with the other, it raises a cluster of bells. The right leg is lifted in a dance step, while the left, toes curled inward for better balance, is rigid. During the Edo period (1603–1868), Kabuki theater programs began at dawn with a dance. In the final of three scenes in this dance, “the bell-tree,” the dancer would shake a wand covered with small bells. Along these lines, the monkey in Mori Sosen’s work lifts the bells in performing the Sanbasō, a dance celebrating the New Year, the first day of the Monkey Year.

Macaques, more than 20 species of *Macaca*, a genus of Old World monkeys mostly found in Asia, occupy a geographic range second only to that of humans in its extent. Their habitat varies from near desert to rainforest, from sea level to snow-covered mountain tops. The Japanese macaque, also known as snow monkey, which has been reported at an elevation of 3,180 m, representing the northernmost nonhuman primate population in the world, has gained some notoriety for visiting a hot spring in Nagano to find comfort from the cold in winter.

Culturally, this monkey has been a metaphor, a polysemic symbol throughout Japanese history―now a mediator between gods and humans, now a scapegoat, now a clown. Because of its unique role as similar to yet different from humans, the Japanese macaque was used to define what it means to be human and alternatively what it means to be a monkey: “human minus three pieces of hair,” to the Japanese. This definition satisfied both the affinity between humans and monkeys and the animal’s local status just below grade.

The monkey’s role as mediator between gods and humans was long lived and well established. It implied possession of supernatural powers, which were often expressed in ritual dances with music. The monkey was believed a guardian that could cure disease in horses and secure good crops as mediator between the Mountain Deity and humans. This status diminished gradually. The monkey was secularized and demoted, becoming the object of ridicule, a scapegoat, for lacking (even if only by 3 pieces of hair) the essence of humanness. Though a monkey dance performance still likely showcases human superiority, the powerful metaphorical presence persists, despite the animal’s virtual disappearance from everyday human contact outside the zoo.

Around the world, the status of macaques and their connection with humans continues to evolve. The Japanese tradition that the monkey was a scapegoat for a human victim of smallpox or of another disease, which persisted for centuries, is no longer held. In more recent times the animals have served instead as models for human disease, providing through their own infections or experimental studies, insight into pathogenic mechanisms, treatments, and vaccine approaches for human infectious agents, among them, hepatitis B, influenza virus, flaviviruses, *Plasmodium* spp. Some infections (HIV-2, *P. knowlesii*) have been transmitted from nonhuman primates to humans, suggesting that the role of these primates as “mediators” persists, but some, including measles and tuberculosis, can go both ways, with infected humans compromising the health of nonhuman primates and because of the infections in the monkeys, an employee health vaccination program was launched, potentially preventing tetanus among workers.

In this issue of the journal, a colony of Japanese macaques saw a mass die-off attributed to severe soil contamination by *Clostridium tetani* in the facility maintaining the animals. In China, *Cryptosporidium* spp., *Giardia duodenalis*, and *Enterocytozoon bieneusi* organisms were detected in free range rhesus monkeys in a popular public park. Most genotypes and subtypes detected were anthroponotic, which indicates that these animals, after becoming infected from exposure to infected humans, may have become reservoirs for human cryptosporidiosis, giardiasis, and microsporidiosis. In Afghanistan, bites from macaques may have exposed US troops and presumably the Afghanis to serious infections, among them rabies, B virus, and tetanus. In Africa, nonhuman primates may be acting as a zoonotic reservoir of *P. vivax* in regions where the human population is almost entirely refractory. If so, with human encroachment into nonhuman primate habitats, the chances of susceptible humans encountering the parasite will increase.

As it lifts up the bells to ring in the New Year 1800, Mori Sosen’s beloved monkey in the flawless kimono continues the age-old dance celebrating our phylogenetic closeness. Because of this closeness, humans and nonhuman primates are susceptible to many of the same infections, minus three pieces of hair or not.
